# Supplementary material for: Metallo-Supramolecular Complexation Behavior of Terpyridine- and Ferrocene-Based Polymers in Solution—A Molecular Hydrodynamics Perspective
Source: Polymers (Basel). 2022 Feb 26;14(5):944. doi: 10.3390/polym14050944 (PMC8912760; doi:10.3390/polym14050944)
Supplement: Supplementary file 1 [file polymers-14-00944-s001.zip › polymers-1589347-supplementary.pdf]

Supporting Information

Metallo-Supramolecular Complexation Behavior of Terpyridine- and Ferrocene-Based Polymers in solution – a Perspective from Molecular Hydrodynamics

Igor Perevyazko,[1] Nina Mikusheva[1], Alexey Lezov[1], Alexander Gubarev[1], Marcel Enke,[2,3] Andreas Winter,[2,3] Ulrich S. Schubert,[2,3]* Nikolay Tsvetkov[1]**

[1] Department of Molecular Biophysics and Polymer Physics, St. Petersburg University, 199034, Universitetskaya nab. 7/9, Saint-Petersburg, Russia

[2] Laboratory of Organic and Macromolecular Chemistry (IOMC), Friedrich Schiller University Jena, Humboldtstr. 10, 07743 Jena, Germany

[3] Jena Center for Soft Matter (JCSM), Friedrich Schiller University Jena, Philosophenweg 7, 07743 Jena, Germany

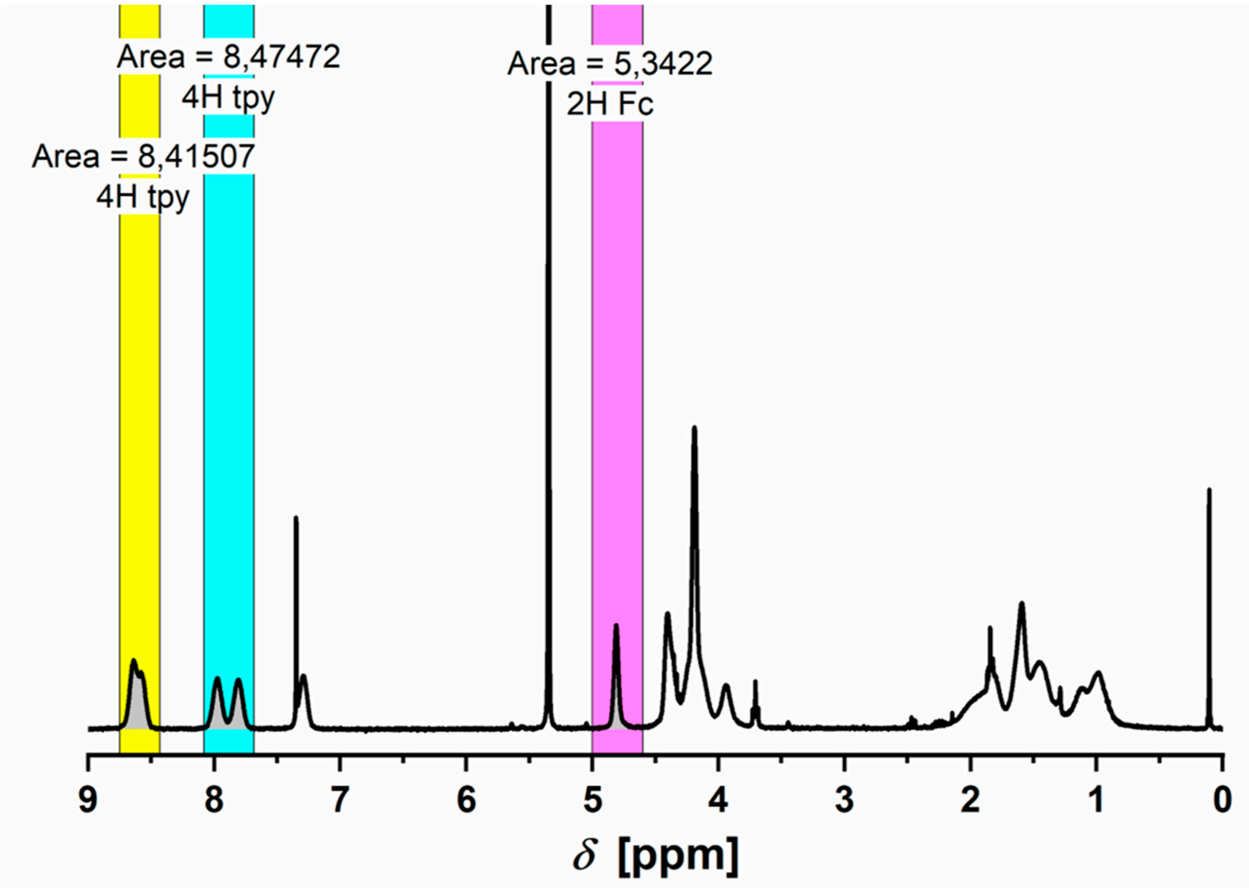

Figure S1. ^{1}HNMR spectra of TerFerCop (right) with corresponding integration data, 300 MHz, $T = 25\ °C$, CD_2Cl_2.

The drops of the TerFerCop solutions/suspensions Eu^{3+} and Pd^{2+} in THF were applied on a silicon wafer and dried. The morphology and sizes of the obtained samples were studied using scanning electron microscope Zeiss Merlin (Carl Zeiss Group, Germany) – for TerFerCop with Eu^{3+} salt and Zeiss Auriga Laser (Carl Zeiss Group, Germany) – for TerFerCop with Pd^{2+} salt. The accelerating voltage was 3 kV. Sizes were obtained using ZEISS SmartSEM software (Carl Zeiss Group, Germany) or SmartTiff V02.00 software (Carl Zeiss Group, Germany).

In Figure S2 images of different areas of the wafer for TerFerCop Eu^{3+} at M/L = 0.5M are shown. It can be seen that sample consists of separate uniform ovoid-like morphology species. The huge aggregates like on part (d) had random shapes.

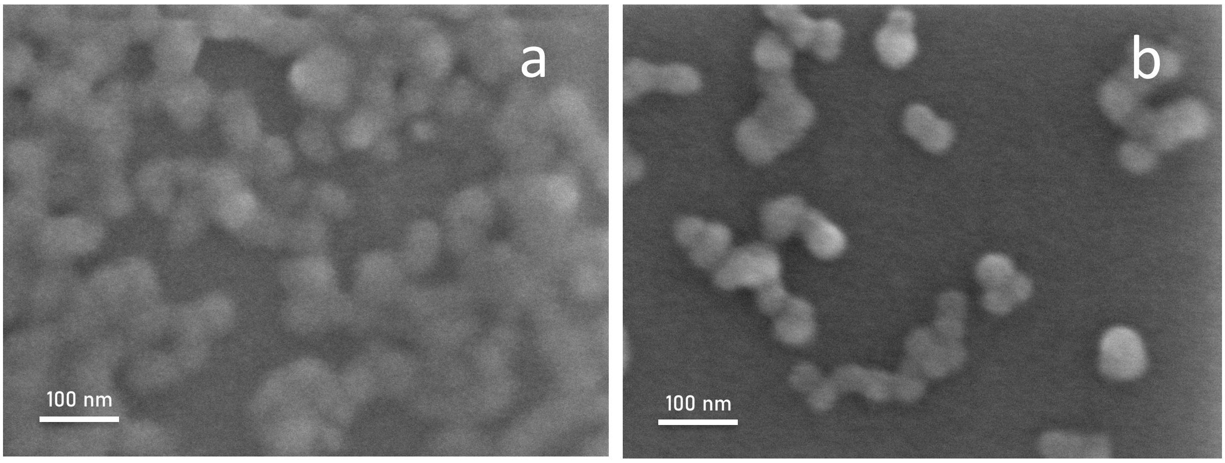

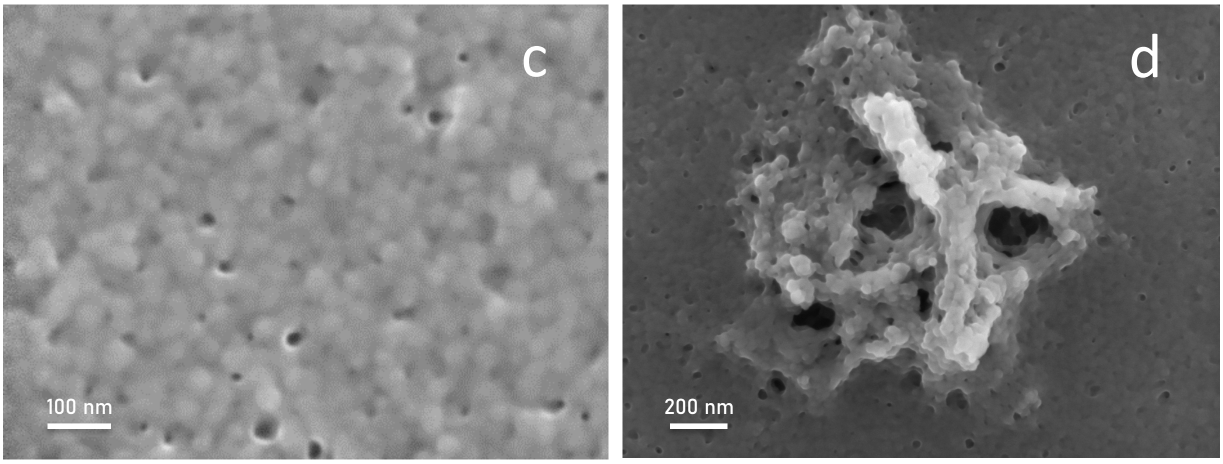

Figure S2. TerFerCop complexes with Eu^{3+} $M/L{\sim}0.5$. (a), (b), (c), (d) correspond to different typical areas on the same wafer. Component size: 40-60 nm

In Figure S3 images of the TerFerCop-Pd^{2+} complexes $M/L{\sim}0.5$, Pd^{2+} are shown. It can be seen that sample consists of separate uniform ovoid-like morphology species too. Average size of components: ~45 nm

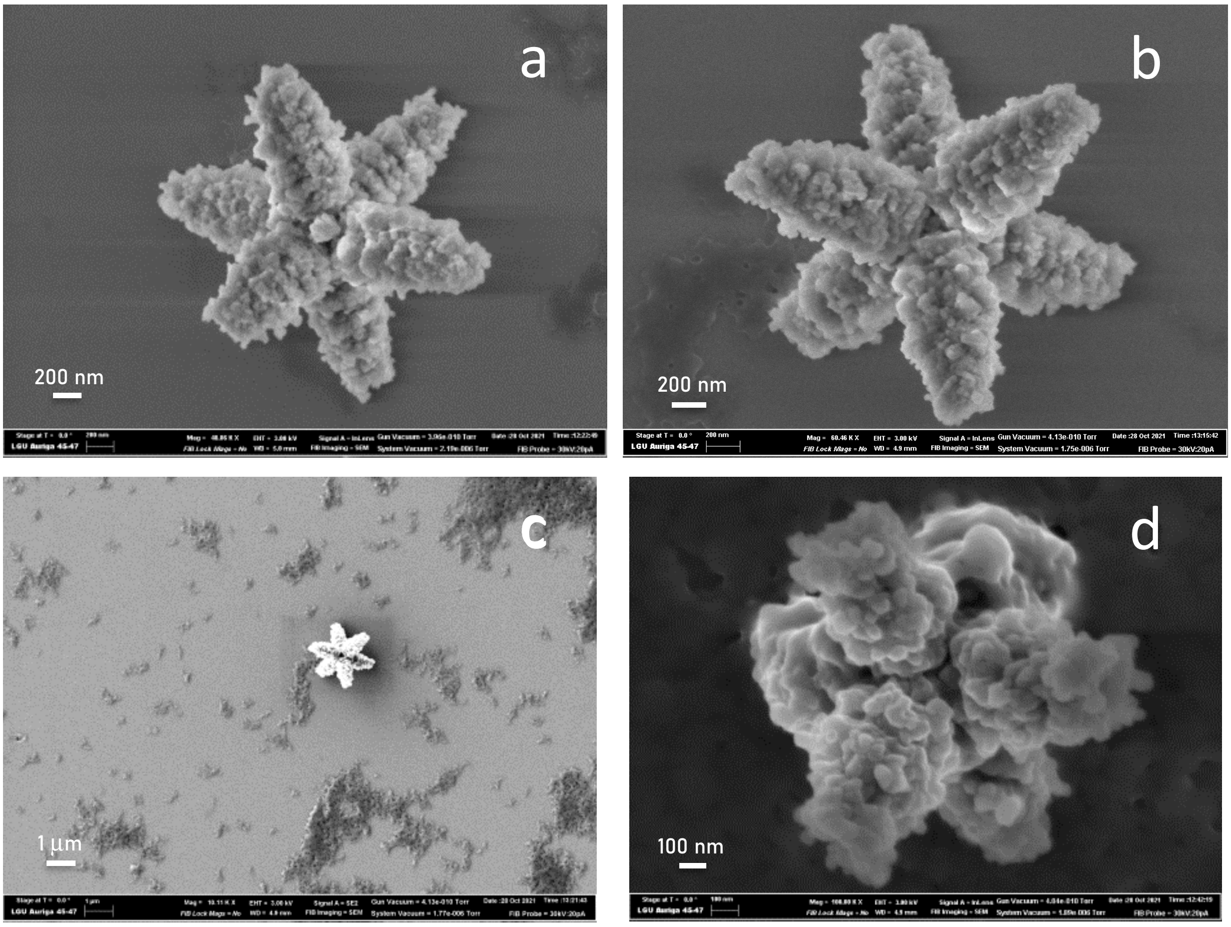

Figure S3. TerFerCop-Pd^{2+} $M/L{\sim}0.4$. (a), (b) correspond to different typical areas on the same wafer. Average size of ovoid-like components: 45 nm.

In Figures S3 and S4 images of different areas of the TerPol-Pd^{2+} systems at $M/L{\sim}0.1M$). It can be seen that the sample consists of separate uniform spherical/globular like morphology species. Observed sizes are presented in the figure descriptions.

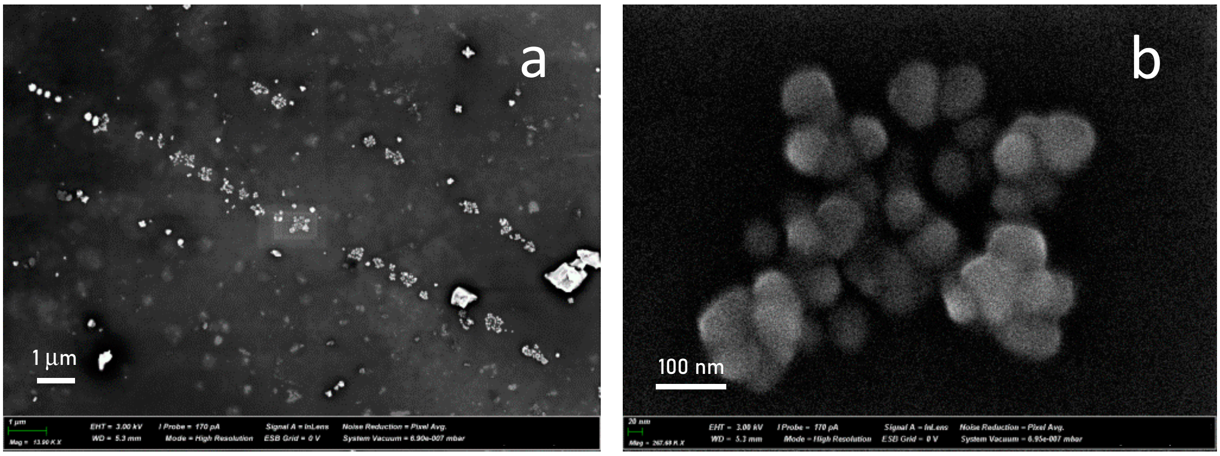

Figure S4. TerPol = 0.05% in THF, Pd^{2+} molar conc = 0,0001M. (a), (b) correspond to the same area on the wafer, in different scale. Size of components (b): 40-60 nm. Size analysis performed using SmartTiff V02.00 software (Carl Zeiss).

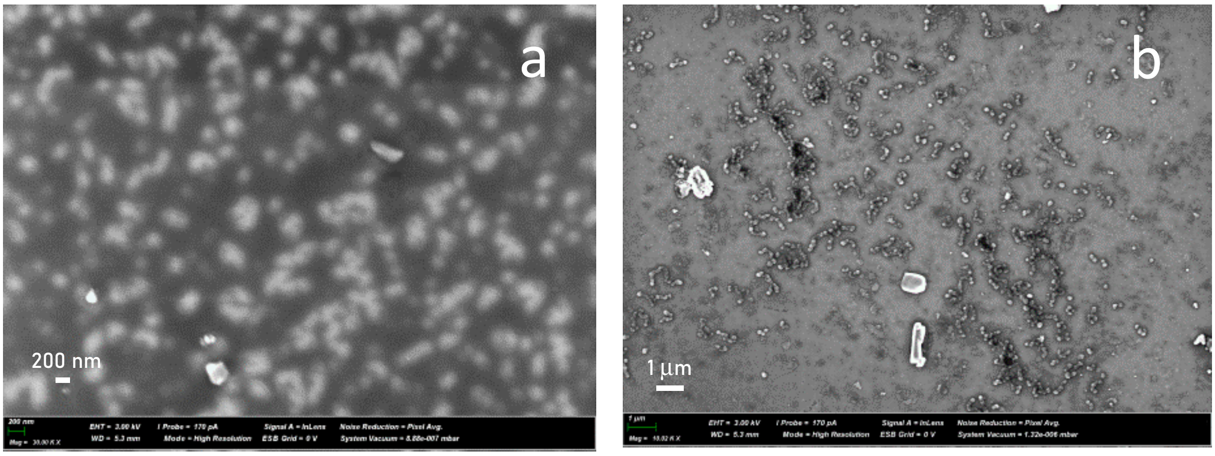

Figure S5. TerPol = 0.05% in THF, Pd^{2+} molar conc = 0,00019M (a) and 0,00042M (b). Maximum size of components on (a): 130 nm. Size of ovoid-like components on (b): 100-160 nm. Size analysis performed using SmartTiff V02.00 software (Carl Zeiss).

Although the direct accordance of sizes of dried particles on a wafer with hydrodynamical sizes in a solution is questionable, we can notice visible growth of particles along with an increasing amount of salt in the polymer solution.

TerFerPol **TerPol**

Eu complexes:

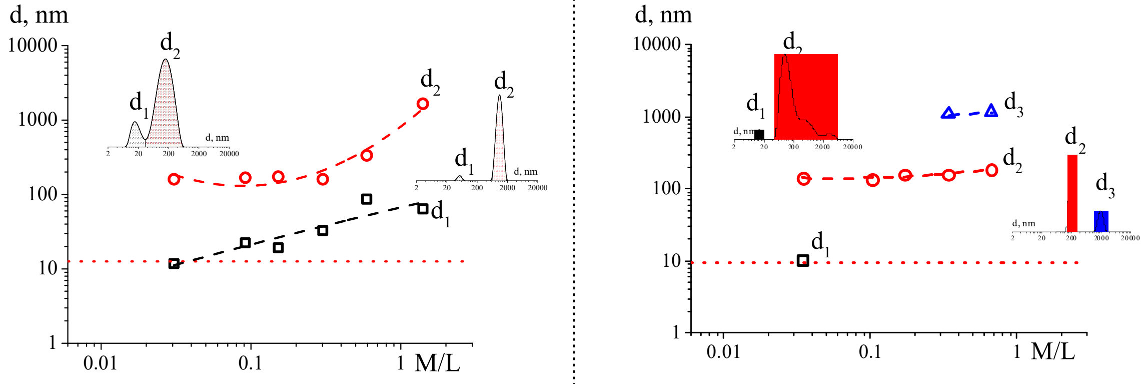

Pd complexes:

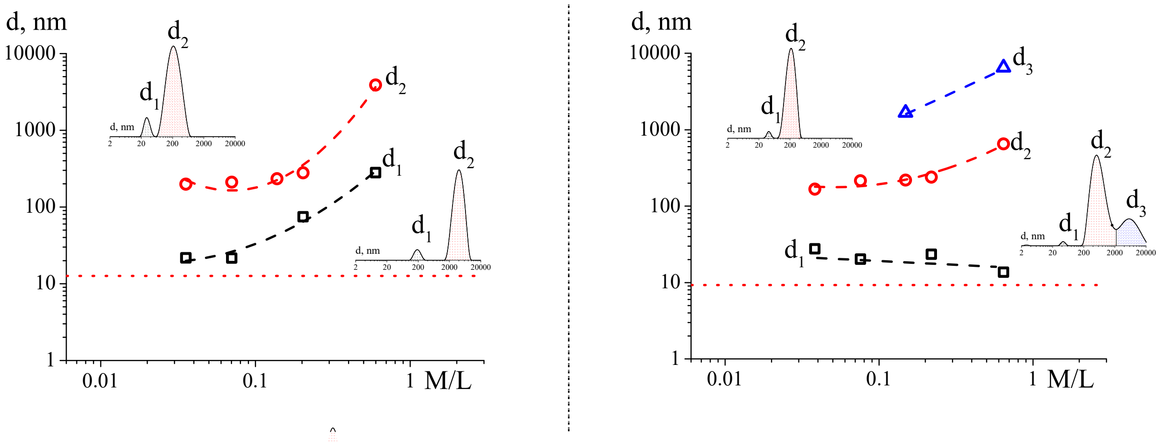

Figure S6. Double logarithm dependences of hydrodynamic diameters d_h obtained by DLS as the function of the Metal to Ligand ratio (M/L). Different symbols correspond to the maxima positions of the observed by DLS distributions (d_1, d_2, d_3). The inserts are DLS distributions of analyzed solutions at minimal and maximal M/L ratio. The red dotted lines correspond to the hydrodynamic diameter of initial copolymer/copolymer in the limit of infinite dilution.
